# Supplementary material for: Prevalence of Antibiotic Resistance and Virulence Genes in Escherichia coli Carried by Migratory Birds on the Inner Mongolia Plateau of Northern China from 2018 to 2023
Source: Microorganisms. 2024 May 26;12(6):1076. doi: 10.3390/microorganisms12061076 (PMC11205581; doi:10.3390/microorganisms12061076)
Supplement: Supplementary file 1 [file microorganisms-12-01076-s001.zip › Table S1-S4.pdf]

Table S1. PCR primers of antimicrobial resistance genes

Genes	Sequence (5′–3′)	Amplicon size (bp)
bla$_{CTX\text{-}M}$	F:ATGTGCAGYACCAGTAARGT<br>R:TGGGTRAARTARGTSACCAGA	544
bla$_{TEM\text{-}1}$	F:ATGAGTATTCAACATTTCCGT<br>R:TTACCAATGCTTAATCAGTGA	861
tet(A)	F:GTAATTCTGAGCACTGTCGC<br>R:CTGCCTGGACAACATTGCTT	956
tet(B)	F:CTCAGTATTCCAAGCCTTTG<br>R:ACTCCCCTGAGCTTGAGGGG	414
tet(M)	F:GTGGACAAAGGTACAACGAG<br>R:CGGTAAAGTTCGTCACACAC	406
sul1	F:CACCGGAAACATCGCTGCA<br>R:AAGTTCCGCCGCAAGGCT	158
sul2	F:CTCCGATGGAGGCCGGTAT<br>R:GGGAATGCCATCTGCCTTGA	190
sul3	F:CCCATACCCGGATCAAGAATAA<br>R:CAGCGAATTGGTGCAGCTACTA	143
floR	F:GGCTTTCGTCATTGCGTCTC<br>R:ATCGGTAGGATGAAGGTGAGGA	650
cmlA	F:TGCCAGCAGTGCCGTTTAT<br>R:CACCGCCCAAGCAGAAGTA	900

Table S2. Resistance spectrum of 91 MDR or ESBL-producing isolates

**Resistance spectrum**	**Number（%）**
AMP-CHL-TET	1（1.1）
AMP-AMC-TET	2（2.2）
ATM-AMC-TET	1（1.1）
PIP-AMC-TET	1（1.1）
SXT-CHL-TET	3（3.4）
CZO-AMP-PIP	2（2.2）
AMP-AMC-CHL-TET	1（1.1）
AMP-PIP-CHL-TET	7（7.9）
AMP-PIP-SXT-TET	9（10.1）
AMP-SAM-CHL-TET	1（1.1）
AMP-SAM-SXT-CHL-TET	1（1.1）
AMP-PIP-SXT-CHL-TET	13（14.6）
AMP-PIP-SAM-CHL-TET	2（2.2）
AMP-PIP-CHL-MXF-TET	1（1.1）
CZO-AMP-AMC-SXT-TET	1（1.1）
CZO-CTX-FEP-AMP-PIP	3（3.4）
GEM-AMP-PIP-SXT-TET	3（3.4）
AMP-PIP-SAM-SXT-CHL-TET	1（1.1）
GEN-AMP-PIP-SXT-CHL-TET	1（1.1）
SXT-CHL-CIP-LVX-MXF-TET	1（1.1）
CZO-CTX-FEP-ATM-AMP-PIP	1（1.1）
AMP-PIP-CHL-CIP-MXF-TET	2（2.2）
AMP-PIP-SXT-CIP-LVX-MXF-TET	8（9.0）
CZO-CTX-AMP-PIP-SXT-CHL-TET	1（1.1）
CZO-CTX-FEP-AMP-PIP-SXT-TET	1（1.1）
GEN-CZO-CTX-FEP-AMP-PIP-SAM-STX	1（1.1）
AMP-PIP-SXT-CHL-CIP-LVX-MXF-TET	2（2.2）
CZO-CTX-ATM-AMP-PIP-SXT-CHL-TET	3（3.4）
CZO-CTX-FEP-ATM-AMP-PIP-CHL-TET	3（3.4）
GEN-CZO-CTX-FEP-AMP-PIP-SXT-CHL-TET	1（1.1）
CZO-CTX-FEP-AMP-PIP-CIP-LVX-MXF-TET	1（1.1）
GEN-AMP-PIP-SAM-SXT-CIP-LVX-MXF-TET	1（1.1）
GEN-AMP-PIP-SXT-CHL-CIP-LVX-MXF-TET	3（3.4）
CZO-CTX-FEP-AMP-PIP-AMC-SAM-SXT-CHL-TET	1（1.1）
CZO-CTX-FEP-AMP-PIP-CHL-CIP-LVX-MXF-TET	1（1.1）
CZO-CTX-FEP-ATM-AMP-PIP-SAM-SXT-CHL-TET	3（3.4）
GEN-CZO-CTX-FEP-ATM-AMP-PIP-SXT-CHL-TET	1（1.1）
GEN-CZO-CTX-FEP-ATM-AMP-PIP-SXT-CHL-CIP-LVX-FEP-TET	1（1.1）
CZO-CAZ-CTX-FEP-ATM-AMP-PIP-AMC-SAM-SXT-CHL-CIP-LVX-FEP-TET	1（1.1）

Table S3. Major ARGs of MDR and ESBL-producing strains

**Main antibiotics**	**Main ARGs**	**Number of virulence factor (%)**
β-lactams	*bla*$_{TEM-1}$	57(62.6)
	bla$_{TEM-176}$	6(6.6)
	bla$_{CTX-M-1}$	1(1.1)
	bla$_{CTX-M-14}$	6(6.6)
	bla$_{CTX-M-15}$	2(2.2)
	bla$_{CTX-M-27}$	3(3.3)
	bla$_{CTX-M-55}$	8(8.8)
	bla$_{CTX-M-65}$	4(4.4)
	bla$_{OXA-10}$	7(7.7)
	bla$_{OXA-1}$	5(5.5)
	bla$_{LAP-2}$	4(4.4)
	bla$_{DHA-1}$	1(1.1)
	bla$_{CMY-2}$	1(1.1)
Tetracycline	*tet(A)*	81(89.0)
	tet(B)	3(3.3)
	tet(M)	5(5.5)
Sulfonamide	*sul1*	15(16.5)
	sul2	48(52.7)
	sul3	14(15.4)
Chloramphenicol	*floR*	51(56.0)
	cmlA1	16(17.6)
	catB3	5(5.5)
	catA1	1(1.1)
	catA2	1(1.1)
Quinolones	*qnrS1*	48(52.7)
	qnrS2	6(6.6)
	qnrB4	1(1.1)
	qnrB7	1(1.1)
Folate metabolism pathway inhibitors	*dfrA14*	42(46.2)
	dfrA12	10(11.0)
	dfrA17	16(17.6)
	dfrA15	2(2.2)
	dfrA7	1(1.1)
Aminoglycoside	*aac(3)-IIa*	1(1.1)
	aac(3)-IId	10(11.0)
	aac(3)-IV	1(1.1)
	aac(6′)-Ib-cr	5(5.5)
	aph(3′)-IIa	4(4.4)

	aph(3′)-Ia	12(13.2)
	aph(3′′)-Ib	43(47.3)
	aadA5	11(12.1)
	aadA1	22(24.2)
	aadA2	10(11.0)
	aadA22	2(2.2)
	aadA2b	2(2.2)
	aph(4)-Ia	1(1.1)
	aph(6)-Id	46(50.5)
Fosfomycin	*fosA7*	7(7.7)
	fosA3	1(1.1)
Rifamycin	*ARR-3*	4(4.4)
	ARR-2	7(7.7)
Antibiotic efflux pump	*qacE*	16(17.6)
	qacL	10(11.0)
Lincomycin	*lnu(F)*	2(2.2)
Macrolides antibiotics	*mph(A)*	11(12.1)
	mef(B)	7(7.7)
External Discharge Pump Complex	*mdf(A)*	12(13.2)

Table S4. Major virulence genes of MDR and ESBL-producing strains

**Virulence genes**	**Virulence factor**	**No.（%）**
astA	EAST1 thermostable toxins	14（15.4）
cnf1	Cytotoxic necrotizing factor	2（2.2）
sen	Enterotoxins	1（1.1）
usp	Urinary pathogenicity-specific proteins	9（9.9）
vat	Vacuolated autotransporter toxin	10（11.0）
ireA	Iron carrier receptor	5（5.5）
iroN		30（33.0）
irp2		21（23.1）
fyuA		20（22.0）
hlyF	Hemolysin F	31（34.1）
