# Supplementary material for: Prevalence of Antibiotic Resistance and Virulence Genes in Escherichia coli Carried by Migratory Birds on the Inner Mongolia Plateau of Northern China from 2018 to 2023
Source: Microorganisms. 2024 May 26;12(6):1076. doi: 10.3390/microorganisms12061076 (PMC11205581; doi:10.3390/microorganisms12061076)
Supplement: Supplementary file 1 [file microorganisms-12-01076-s001.zip › Table S5.pdf]

Table S5 Background information on the 91 MDR or ESBL *Escherichia coli* strains framework diagrams

	Strain	Year	location	ESBL	Resistance pattern	-Resistance number	Resistance genes	Virulence genes	MLST	Serotype	Plasmid	ExPEC	APEC	Transconjugants
1	G44	2018spring	Ningxia	-	AMP-PIP-SXT-TET	3R	*blaTEM-1，tet(A)*	*lpfA, ompT, papC, terC*	1246	O166:H49	IncY	N	N	N
2	210311G10	2021spring	Ningxia	+	GEN-CZO-CTX-FEP-ATM-AMP-PIP-SXT-CHL-CIP-LVX MXF-TET	9R	*aadA1, aadA22, aph(3')-Ia, aac(3)-IIa, blaCTX-M-55, qacL, sitABCD, mef(B), lnu(F), floR, sul3, tet(A), dfrA14*	*AslA, anr, cma, csgA, cvaC, fimH, gad, hlyF, iucC, iutA, nlpI, ompT, sitA, terC, traJ, traT, yehA, yehB, yehC, yehD*	206	ND	IncFIB、IncFIC、IncY	N	N	Y
3	210311G40	2021spring	Ningxia		CZO-CTX-AMP-PIP-SXT-CHL-TET	6R	*aadA1, blaCTX-M-65, qacE, floR, qnrS2, sul1, tet(A)*	*csgA, fdeC, fedB, fimH, gad, hlyE, lpfA, nlpI, terC, yehA, yehB, yehC, yehD*	2144	O170:H49	IncY	N	N	N
4	210326G11	2021spring	Ningxia	-	AMP-PIP-SAM-CHL-TET	4R	*aph(3')-Ia, aadA1, blaOXA-10, blaTEM-176, cmlA1, floR, qnrS1, ARR-2, tet(A), dfrA14*	*cia, csgA, fdeC, fimH, gad, hlyE, iss, lpfA, nlpI, ompT, papC, terC, traJ, traT, yehA, yehB, yehC, yehD*	5891	O122ab:H19	IncFIB、IncI1-I、IncX1	N	N	N
5	210326G14	2021spring	Ningxia	+	CZO-CTX-ATM-AMP-PIP-SXT-CHL-TET	7R	*aadA1, blaCTX-M-65, qacE, floR, qnrS2, sul1, tet(A)*	*csgA, fdeC, fedB, fedC, fimH, gad, hlyE, lpfA, nlpI, terC, yehA, yehB, yehC, yehD*	2144	O170:H49	IncY	N	N	N
6	210326Y14	2021spring	Ningxia	+	CZO-CTX-ATM-AMP-PIP-SXT-CHL-TET	7R	*aadA1, blaCTX-M-65, qacE, floR, qnrS2, sul1, tet(A)*	*csgA, fdeC, fedB, fedC, fimH, gad, hlyE, lpfA, nlpI, terC, yehA, yehB, yehC, yehD*	2144	O170:H49	IncY	N	N	N
7	210326G29	2021spring	Ningxia	+	CZO-CTX-FEP-AMP-PIP-CHL-CIP-LVX-MXF-TET	6R	*aph(6)-Id, aph(3'')-Ib, blaCTX-M-14, sitABCD, fosA7, floR, sul2, tet(A)*	*anr, csgA, cvaC, etsC, fdeC, fimH, fyuA, gad, hlyE, hlyF, hra, iroN, irp2, iss, iucC, iutA, lpfA, mchF, nlpI, ompT, papA_F19, shiB, sitA, terC, traJ, traT, tsh, yehA, yehB, yehC, yehD*	602	O103:H21	IncFIA、IncFIB、IncFIC、IncI1-I	Y	Y	N
8	210326G31	2021spring	Ningxia	-	AMP-PIP-SXT-CHL-TET	4R	*aph(6)-Id, aph(3'')-Ib, blaTEM-1, sitABCD, floR, qnrS1, sul2, tet(A), dfrA14*	*AslA, anr, astA, cba, cea, chuA, cia, cma, csgA, dhaK, fdeC, fimH, fyuA, gad, ibeA, irp2, iss, kpsE, nlpI, ompT, papC, pic, sitA, terC, traJ, traT, usp, vat, yehA, yehB, yehC, yehD, yfcV*	355	O120:H5	IncFIB、IncFII、p0111	N	Y	N
9	21ZN18dc	2021autumn	Ningxia	-	CZO-AMP-AMC-SXT-TET	5R	*aph(6)-Id, aadA5, aph(3'')-Ib, blaDHA-1, mph(A), qnrB4, sul1, sul2, tet(A), dfrA17, dfrA7*	*AslA, afaD, air, chuA, csgA, eilA, espY2, fdeC, fimH, fyuA, hha, hha, hlyE, irp2, iss, kpsE, kpsMII_K1, neuC, nlpI, terC, yehB, yehC, yehD*	349	O86:H2	IncQ1	Y	N	N
10	21ZN21dc	2021autumn	Ningxia	+	CZO-CTX-FEP-ATM-AMP-PIP-CHL-TET	6R	*aph(6)-Id, aph(3')-IIa, aph(3'')-Ib, blaTEM-1B, blaCTX-M-55, floR, qnrS1, sul2, tet(A), tet(M)*	*csgA, fdeC, fimH, gad, hha, hlyE, iss, lpfA, nlpI, ompT, papC, terC, traJ, traT, yehA, yehB, yehC, yehD*	1844	O8:H49	IncFII、p0111	N	N	Y
11	21ZN23dc	2021autumn	Ningxia	-	AMP-PIP-CHL-TET	3R	*aph(6)-Id, aph(3'')-Ib, blaTEM-1, sitABCD, floR, qnrS1, sul2, tet(A)*	*csgA, fdeC, fimH, gad, hlyE, iss, lpfA, nlpI, ompT, sitA, terC, yehA, yehB, yehC, yehD*	162	O8:H28	ND	N	N	N

12	21ZN33dc	2021autumn	Ningxia	+	CZO-CTX-FEP-AMP-PIP-SXT-TET	5R	*aph(6)-Id, aadA5, aph(3'')-Ib, blaCTX-M-27, qacE, sitABCD, mph(A), sul1, sul2, tet(A), dfrA17*	*AslA, chuA, csgA, eilA, fdeC, fimH, fyuA, gad, hha, hlyE, iha, irp2, iss, iucC, iutA, kpsE, lpfA, nlpI, ompT, papA_fsiA_F16, papC, sat, senB, sitA, terC, traJ, traT, yehA, yehB, yehC, yehD*	69	ND	Col156、IncB/O/K/Z、IncFIB、IncFII	Y	N	Y
13	21ZN35dc	2021autumn	Ningxia	-	AMP-PIP-SXT-TET	3R	*aph(6)-Id, aph(3'')-Ib, blaTEM-1, sitABCD, qnrS1, sul2, tet(A), dfrA14*	*AslA, anr, chuA, csgA, cvaC, dhaK, etsC, fdeC, fimH, fyuA, hlyF, ibeA, iroN, irp2, iss, iucC, iutA, kpsE, kpsMII_K1, mchF, neuC, nlpI, ompT, sitA, terC, traT, tsh, usp, vat, yehA, yehB, yehC, yehD, yfcV*	569	O134:H31	IncFIA、IncFIB、IncFIC	Y	Y	N
14	21ZN40dc	2021autumn	Ningxia	-	AMP-PIP-SAM-CHL-TET	4R	*aph(6)-Id, aadA1, blaOXA-10, blaTEM-1, cmlA1, floR, qnrS1, ARR-2, tet(A), tet(M), dfrA14*	*csgA, fdeC, fimH, gad, hlyE, nlpI, terC, yehA, yehB, yehC, yehD*	398	O182:H40	IncN、IncY、	N	N	N
15	21ZN41dc	2021autumn	Ningxia	-	AMP-PIP-SXT-CHL-TET	4R	*blaTEM-1, mef(B), floR, qnrS1, sul3, tet(A), dfrA14*	*csgA, fimH, gad, hlyE, iss, lpfA, nlpI, ompT, terC, tia, yehA, yehB, yehC, yehD*	1720	O29:H10	IncX1	N	N	N
16	21ZN43dc	2021autumn	Ningxia	-	AMP-PIP-CHL-TET	3R	*blaTEM-1, floR, qnrS1, tet(A), dfrA14*	*csgA, fdeC, fimH, gad, hlyE, lpfA, nlpI, terC, yehA, yehB, yehC, yehD*	297	O149:H8	IncY	N	N	N
17	21ZN49dc	2021autumn	Ningxia	-	GEN-AMP-PIP-SXT-CHL-TET	5R	*aph(6)-Id, aph(3')-IIa, aac(3)-IId, aadA5, aac(6')-Ib-cr, aph(3'')-Ib, blaOXA-1, qacE, mph(A), catB3, floR, qnrS2, ARR-3, sul1, sul2, tet(A), dfrA17*	*AslA, anr, csgA, fdeC, fimH, gad, hlyE, iss, nlpI, terC, traT, yehA, yehB, yehC, yehD,*	1286	O16:H32	IncFII、IncX1、	N	N	N
18	21ZN56dc	2021autumn	Ningxia	-	AMP-PIP-SXT-TET	3R	*aph(6)-Id, aph(3'')-Ib, blaTEM-1, blaLAP-2, qnrS1, sul2, tet(A), dfrA14*	*clpK1, csgA, fdeC, gad, hlyE, nlpI, terC, yehA, yehB, yehC, yehD*	181	O51:H21	IncFIB	N	N	N
19	21ZN57dc	2021autumn	Ningxia	+	CZO-CAZ-CTX-FEP-ATM-AMP-PIP-AMC-SAM-SXT-CHL-CIP-LVX-MXF-TET	9R	*aph(6)-Id, aph(3'')-Ib, blaCMY-2, blaTEM-1, blaCTX-M-27, sitABCD, fosA7, catA1, sul2, tet(B), dfrA17*	*anr, astA, cib, csgA, etsC, fdeC, fimH, fyuA, gad, hlyE, hlyF, iroN, irp2, iss, iucC, iutA, lpfA, nlpI, ompT, shiB, sitA, terC, traJ, traT, yehA, yehB, yehC, yehD*	602	O9:H9	IncFIB、IncFIC、IncI1-I、IncQ1	N	N	N
20	21ZN60dc	2021autumn	Ningxia	+	CZO-CTX-ATM-AMP-PIP-SXT-CHL-TET	7R	*aph(6)-Id, aph(3'')-Ib, blaTEM-1B, blaCTX-M-65, sitABCD, floR, qnrS2, sul2, tet(A), dfrA14*	*csgA, fdeC, fimH, gad, hlyE, lpfA, nlpI, sitA, terC, yehA, yehB, yehC, yehD*	1049	O8:H10	p0111	N	N	N
21	21ZN70dc	2021autumn	Ningxia	-	AMP-PIP-SXT-CHL-CIP-LVX-MXF-TET	5R	*aph(6)-Id, aph(3')-Ia, aph(3'')-Ib, blaTEM-1, sitABCD, floR, sul2, sul3, tet(A), dfrA14*	*anr, cma, csgA, cvaC, fdeC, fimH, gad, hha, hlyE, hlyF, iroN, iss, iucC, iutA, lpfA, nlpI, ompT, sitA, terC, traJ, traT, yehA, yehB, yehC, yehD*	115	O23:H51	IncFIB、IncFIC	N	N	N
22	21ZN71dc	2021autumn	Ningxia	+	CZO-CTX-FEP-ATM-AMP-PIP-CHL-TET	6R	*aph(6)-Id, aph(3')-IIa, aph(3'')-Ib, blaTEM-1, blaCTX-M-55, floR, qnrS1, sul2, tet(A), tet(M)*	*csgA, fdeC, fimH, gad, hha, hlyE, iss, lpfA, nlpI, ompT, papC, terC, traJ, traT, yehA, yehB, yehC, yehD*	1844	O8:H49	IncFII、p0111	N	N	Y
23	21ZN94dc	2021autumn	Ningxia	-	AMP-PIP-SXT-CHL-CIP-LVX-MXF-TET	5R	*aph(6)-Id, aph(3'')-Ib, blaTEM-1, sitABCD, floR, sul2, tet(A), dfrA14*	*anr, astA, csgA, cvaC, etsC, fdeC, fimH, gad, hha, hlyE, hlyF, hra, iroN,*	162	O160:H16	IncFIB、IncFIC	Y	Y	N

								iss, iucC, iutA, lpfA, nlpI, ompT, papC, sitA, terC, traJ, traT, yehA, yehB, yehC, yehD						
24	21ZN95dc	2021autumn	Ningxia	+	CZO-CTX-FEP-ATM-AMP-PIP-CHL-TET	6R	aph(6)-Id, aph(3')-IIa, aph(3'')-Ib, blaTEM-1, blaCTX-M-55, floR, qnrS1, sul2, tet(A), tet(M)	csgA, fdeC, fimH, gad, hha, hlyE, iss, lpfA, nlpI, ompT, papC, terC, traJ, traT, yehA, yehB, yehC, yehD	1844	O8:H49	IncFII、p0111	N	N	Y
25	21ZN96dc	2021autumn	Ningxia	-	AMP-PIP-SXT-CHL-TET	4R	aph(6), aadA1, aph(3'')-Ib, blaTEM-1, qacE, sitABCD, floR_2, sul2, tet(A)	anr, csgA, fdeC, fimH, hlyE, hlyF, iroN, iss, lpfA, nlpI, ompT, papA, shiA, sitA, terC, tia, traJ, traT, yehA, yehB, yehC, yehD	ND	O155:H11	IncFIB、IncFII、p0111	N	N	N
26	21ZN98dc	2021autumn	Ningxia	-	AMP-PIP-CHL-TET	3R	aph(3')-Ia, blaTEM-176, sitABCD, floR, qnrS1, tet(A), dfrA14	csgA, fdeC, fimH, gad, hlyE, iss, lpfA, nlpI, ompT, sitA, terC, yehA, yehB, yehC, yehD	906	O150:H8	IncX1	N	N	N
27	21ZN101dc	2021autumn	Ningxia	-	AMP-PIP-CHL-TET	3R	aph(3')-Ia, blaTEM-176, sitABCD, floR, qnrS1, tet(A), dfrA14	csgA, fdeC, fimH, gad, hlyE, iss, lpfA, nlpI, ompT, sitA, terC, yehA, yehB, yehC, yehD	906	O150:H8	IncX1	N	N	N
28	21ZN102dc	2021autumn	Ningxia	-	AMP-PIP-SXT-TET	3R	aph(6)-Id, aph(3'')-Ib, blaTEM-1, sitABCD, qnrS1, sul2, tet(A), dfrA14	anr, csgA, cvaC, etsC, fdeC, fimH, fyuA, gad, hlyE, hlyF, iroN, irp2, iss, iucC, iutA, lpfA, mchF, nlpI, ompT, sitA, sitA, terC, traT, tsh, yehA, yehB, yehC, yehD	297	O54:H8	IncFIA、IncFIB、IncFIC	N	Y	N
29	21ZN116dc	2021autumn	Ningxia	-	AMP-PIP-SXT-TET	3R	aph(6)-Id, aph(3')-Ia, aadA1, blaTEM-1, qacL, sitABCD, qnrS1, sul3, tet(A), tet(M), dfrA17	anr, cma, csgA, cvaC, fdeC, fimH, gad, hlyE, hlyF, hra, iroN, iss, iucC, iutA, lpfA, nlpI, ompT, shiA, sitA, terC, tia, traJ, traT, yehA, yehB, yehC, yehD	616	O21:H45	IncFIB、IncFIC、IncX1、p0111	N	N	N
30	21ZN118dc	2021autumn	Ningxia	-	SXT-CHL-TET	3R	aph(6)-Id, aph(3'')-Ib, blaLAP-2, floR_2, qnrS1, sul2, tet(A), dfrA14	AslA, csgA, fdeC, fimH, gad, hlyE, nlpI, terC, yehA, yehB, yehC, yehD	ND	O15:H11	p0111	N	N	N
31	21ZN120dc	2021autumn	Ningxia	-	SXT-CHL-CIP-LVX-MXF-TET	4R	aadA5, qacE, mph(A), floR, sul1, sul2, tet(A), dfrA17	F17A, F17C, F17D, F17G, csgA, eltIIAB-c6, fdeC, fimH, gad, hlyE, iss, lpfA, nlpI, ompT, terC, traT, yehA, yehB, yehC, yehD	2973	O65:H16	IncFIB、IncFII、IncY	N	N	N
32	21ZN122dc	2021autumn	Ningxia	-	SXT-CHL-TET	3R	aadA2, aadA1, qacL, cmlA1, floR, sul3, tet(A), dfrA12	csgA, fdeC, gad, hha, hlyE, lpfA, nlpI, ompT, terC, yehA, yehB, yehC, yehD	155	O64:H20	IncI、p0111	N	N	N
33	21ZN127dc	2021autumn	Ningxia	-	AMP-PIP-CHL-TET	3R	aph(3')-Ia, blaTEM-176, sitABCD, floR, qnrS1, tet(A), dfrA14	AslA, csgA, fdeC, fimH, gad, hha, hlyE, kpsE, kpsMIII_K96, nlpI, sitA, terC, yehA, yehB, yehC, yehD	10	O107:H10	IncX1	N	N	N
34	21ZN129dc	2021autumn	Ningxia	-	AMP-PIP-SXT-CHL-TET	4R	aadA1, aph(3')-Ia, aadA2b, blaTEM-1, qacL, mef(B), cmlA1, qnrS1, sul3, tet(A), dfrA15	csgA, fdeC, fimH, gad, hlyE, lpfA, nlpI, terC, yehA, yehB, yehC, yehD	155	O109:H12	p0111	N	N	N
35	21ZN130dc	2021autumn	Ningxia	-	AMP-PIP-SXT-CHL-TET	4R	aadA1, aph(3')-Ia, aadA2b, blaTEM-1, qacL, mef(B), cmlA1, qnrS1, sul3, tet(A), dfrA15	csgA, fdeC, fimH, gad, hlyE, lpfA, nlpI, terC, yehA, yehB, yehC, yehD	155	O109:H12	p0111	N	N	N

36	21ZN132dc	2021autumn	Ningxia	-	AMP-PIP-CHL-TET	3R	*aph(3')-Ia, blaTEM-176, sitABCD, floR, qnrS1, tet(A), dfrA14*	*AslA, csgA, fdeC, fimH, gad, hha, hlyE, kpsE, kpsMIII_K96, nlpI, sitA, terC, yehA, yehB, yehC, yehD*	10	O107:H10	IncX1	N	N	N
37	21PLY25dc	2021autumn	Ningxia	-	AMP-SAM-SXT-CHL-TET	5R	*aadA1, blaOXA-10, sitABCD, cmlA1, floR, qnrS2, ARR-2, sul2, tet(B), dfrA14*	*csgA, fdeC, fimH, gad, hlyE, lpfA, nlpI, sitA, terC, yehA, yehB, yehC, yehD*	2526	ND	IncI1-I、IncY	N	N	N
38	21QTXY41dc	2021autumn	Ningxia	-	AMP-PIP-SXT-TET	3R	*aph(6)-Id, blaTEM-1, qnrS1, sul3, tet(A), dfrA14*	*AslA, air, chuA, csgA, cvaC, eilA, fdeC, fimH, gad, hlyE, iss, kpsE, kpsMII, lpfA, mchF, nlpI, terC, traT, yehA, yehB, yehC, yehD*	720	ND	IncB/O/K/Z、IncX1、	N	N	N
39	21THG229dc	2021autumn	Ningxia	-	AMP-CHL-TET	3R	*aadA1, blaOXA-10, cmlA1, floR, qnrS1, ARR-2, tet(A), dfrA14*	*AslA, csgA, fimH, gad, hlyE, hra, nlpI, ompT, terC, yehA, yehB, yehC, yehD*	2705	ND	IncFIB	N	N	N
40	21THY231dc	2021autumn	Ningxia	-	GEN-AMP-PIP-SAM-SXT-CIP-LVX-MXF-TET	6R	*aph(6)-Id, aac(3)-IId, aadA5, aac(6')-Ib-cr, aph(3'')-Ib, blaOXA-1, blaTEM-1, sitABCD, mph(A), catB3, sul2, tet(B), dfrA17*	*anr, csgA, fdeC, fimH, gad, hlyE, iss, nlpI, sitA, terC, traT, yehA, yehB, yehC, yehD*	90	H9:O8	IncFIA、IncFIB、IncFII、IncQ1、p0111	N	N	N
41	22ZNY81dc	2022spring	Ningxia	+	CZO-CTX-FEP-ATM-AMP-PIP-SAM-SXT-CHL-TET	8R	*aph(6), aadA5, aac(6')-Ib-cr, aph(3'')-Ib, blaOXA-1, blaCTX-M-55, qacE, sitABCD, fosA7, catB3, floR, ARR-3, sul1, sul2, tet(A), dfrA17*	*anr, csgA, cvaC, etsC, fdeC, fimH, gad, hha, hlyE, hlyF, iroN, iss, iucC, iutA, lpfA, mchF, nlpI, ompT, papA, papC, shiA, shiB, sitA, terC, tia, traJ, traT, yehA, yehB, yehC, yehD*	ND	O54:H7	IncFIB、IncFIC	Y	N	N
42	22ZNG-108dc	2022spring	Ningxia	+	CZO-CTX-FEP-ATM-AMP-PIP-SAM-SXT-CHL-TET	8R	*aph(6)-Id, aadA5, aac(6')-Ib-cr, aph(3'')-Ib, blaOXA-1, blaCTX-M-55, qacE, sitABCD, fosA7, catB3, floR, ARR-3, sul2, tet(A), dfrA17*	*anr, csgA, cvaC, etsC, fdeC, fimH, gad, hha, hlyE, hlyF, iroN, iss, iucC, iutA, lpfA, mchF, nlpI, ompT, papA, papC, shiA, shiB, sitA, terC, tia, traJ, traT, yehA, yehB, yehC, yehD*	6316	O54:H7	IncFIB、IncFIC	Y	N	N
43	22ZNG-131dc	2022spring	Ningxia	+	CZO-CTX-FEP-ATM-AMP-PIP-SAM-SXT-CHL-TET	8R	*aph(6)-Id, aadA5, aac(6')-Ib-cr, aph(3'')-Ib, blaOXA-1, blaCTX-M-55, qacE, sitABCD, fosA7, catB3, floR, ARR-3, sul1, sul2, tet(A), dfrA17*	*anr, csgA, cvaC, etsC, fdeC, fimH, gad, hha, hlyE, hlyF, iroN, iss, iucC, iutA, lpfA, mchF, nlpI, ompT, papA, papC, shiA, shiB, sitA, terC, tia, traJ, traT, yehA, yehB, yehC, yehD*	6316	O54:H7	IncFIB、IncFIC	Y	N	N
44	22ZNG-196dc	2022spring	Ningxia	+	CZO-CTX-FEP-AMP-PIP-CIP-LVX-MXF-TET	5R	*aph(6)-Id, aph(3'')-Ib, blaCTX-M-27, sitABCD, sul2, tet(A)*	*AslA, cea, chuA, csgA, fdeC, fimH, fyuA, gad, irp2, iss, iucC, iutA, nlpI, ompT, papA, sat, sitA, terC, traT, usp, yehA, yehB, yehC, yehD, yfcV*	131	O25:H4	Col(BS512)、IncB/O/K/Z、IncFIA、IncFIB	Y	N	N
45	22ZNG-214dc	2022spring	Ningxia	-	AMP-PIP-CHL-CIP-MXF-TET	4R	*blaTEM-1C, sitABCD, floR, qnrS1, tet(A), dfrA14*	*AslA, anr, chuA, cma, csgA, etsC, fdeC, fimH, gad, hlyF, iucC, iutA, lpfA, nlpI, ompT, sitA, terC, traT, yehA, yehB, yehC, yehD*	3346	O149:H34	IncFIB、IncFIC	N	N	N
46	22ZNG-217dc	2022spring	Ningxia	-	AMP-PIP-CHL-CIP-MXF-TET	4R	*blaTEM-1C, sitABCD, floR, qnrS1, tet(A), dfrA14*	*AslA, anr, chuA, cma, csgA, etsC, fdeC, fimH, gad, hlyF, iucC, iutA, lpfA, nlpI, ompT, sitA, terC, traT, yehA, yehB, yehC, yehD*	3346	O149:H34	IncFIB、IncFIC、	N	N	N

47	22ZNY217dc	2022spring	Ningxia	-	AMP-PIP-CHL-MXF-TET	4R	*blaTEM-1, sitABCD, floR, qnrS1, tet(A), dfrA14*	*AslA, anr, chuA, cma, csgA, etsC, fdeC, fimH, gad, hlyF, iucC, iutA, lpfA, nlpI, ompT, sitA, terC, traT, yehA, yehB, yehC, yehD*	3346	O149:H34	IncFIB、IncFIC	N	N	N
48	22NPL-G111	2022autumn	Ningxia	-	AMP-PIP-SXT-CHL-TET	4R	*aadA5, blaTEM-1, sitABCD, floR, qnrS1, sul2, tet(A), dfrA17, dfrA14*	*anr, cma, csgA, cvaC, etsC, fdeC, fimH, gad, hlyE, hlyF, iroN, iss, iucC, iutA, lpfA, nlpI, ompT, sitA, terC, traJ, traT, yehA, yehB, yehC, yehD*	192	O178:H28	IncFIB、IncFIC、IncY	N	N	N
49	22NPL-Y117	2022autumn	Ningxia	-	AMP-PIP-SXT-CHL-TET	4R	*aph(6)-Id, aph(3'')-Ib, blaTEM-1, sitABCD, floR, qnrS1, sul2, tet(A), dfrA14,*	*AslA, air, anr, chuA, csgA, cvaC, eilA, etsC, fdeC, fimH, fyuA, gad, hha, hlyE, hlyF, iroN, irp2, iss, iucC, iutA, kpsE, kpsMIII_K96, lpfA, mchF, nlpI, ompT, papA, shiA, sitA, terC, tia, traT, tsh, yehA, yehB, yehC, yehD*	11188	O15:H18	IncFIA、IncFIB、IncFIC、	Y	Y	N
50	22NPL-G122	2022autumn	Ningxia	-	AMP-AMC-TET	3R	*tet(A)*	*AslA, astA, csgA, fimH, gad, hlyE, nlpI, terC, traT, yehA, yehB, yehC, yehD*	48	ND	IncFIB、IncY	N	N	N
51	22NPL-G140	2022autumn	Ningxia	-	AMP-PIP-CHL-TET	3R	*blaTEM-1, sitABCD, floR, qnrS1, tet(A), dfrA14*	*anr, cba, cma, csgA, cvaC, etsC, fdeC, fimH, gad, hlyE, hlyF, iroN, iss, iucC, iutA, lpfA, nlpI, ompT, papC, sitA, terC, traJ, traT, yehA, yehB, yehC, yehD*	8146	O32:H8	IncFIA、IncFIB、IncFIC、IncFII	Y	N	N
52	22NPL-G163	2022autumn	Ningxia	-	AMP-AMC-TET	3R	*tet(A)*	*AslA, astA, csgA, fimH, gad, hlyE, nlpI, terC, traT, yehA, yehB, yehC, yehD*	48	ND	ND	N	N	N
53	23THY17EC	2023spring	Ningxia	-	GEN-AMP-PIP-SXT-CHL-CIO-LVX-MXF-TET	6R	*aph(6)-Id, aac(3)-IId, aadA2, aph(3'')-Ib, blaTEM-1B, qacE, sitABCD, mph(A), floR, sul1, sul2, tet(A), dfrA12*	*AslA, anr, capU, csgA, cvaC, fimH, fyuA, gad, hlyE, hlyF, ireA, iroN, irp2, iss, iucC, iutA, kpsE, kpsMII_K5, nlpI, ompT, papA, shiA, sitA, terC, tia, traJ, yehA, yehB, yehC, yehD*	93	O5:H4	IncFIB、IncFIC	Y	N	N
54	23THY39EC	2023spring	Ningxia	-	AMP-PIP-SXT-CHL-TET	4R	*aadA1, aadA2, blaTEM-1, qacL, mef(B), cmlA1, floR, qnrS1, sul3, tet(A), dfrA12*	*csgA, fdeC, fimH, gad, hlyE, iss, lpfA, nlpI, papC, terC, yehA, yehB, yehC, yehD*	351	O18:H7	IncY	N	N	N
55	23THY40EC	2023spring	Ningxia	-	AMP-PIP-SXT-CHL-TET	4R	*aadA1, aadA2, blaTEM-1, qacL, mef(B), cmlA1, floR, qnrS1, sul3, tet(A), dfrA12*	*csgA, fdeC, fimH, gad, hlyE, iss, lpfA, nlpI, papC, terC, yehA, yehB, yehC, yehD*	351	O18:H7	IncY	N	N	N
56	23THY47EC	2023spring	Ningxia	-	AMP-PIP-SXT-CHL-TET	4R	*aadA1, aadA2, blaTEM-1, qacL, mef(B), cmlA1, floR, qnrS1, sul3, tet(A), dfrA12*	*csgA, fdeC, fimH, gad, hlyE, iss, lpfA, nlpI, papC, terC, yehA, yehB, yehC, yehD*	351	O18:H7	IncY	N	N	N
57	23THG52EC	2023spring	Ningxia	-	AMP-PIP-SXT-CHL-TET	4R	*aadA1, aadA2, blaTEM-1, qacL, cmlA, floR, qnrS1, sul3, tet(A), dfrA12*	*csgA, fdeC, fimH, gad, hlyE, iss, lpfA, nlpI, papC, terC, yehA, yehB, yehC, yehD*	351	O18:H7	IncY	N	N	N
58	23THG85EC	2023spring	Ningxia	-	GEN-AMP-PIP-SXT-CHL-CIP-LVX-MXF-TET	6R	*aph(6)-Id, aac(3)-IId, aadA2, aph(3'')-Ib, blaTEM-1, qacE, sitABCD, mph(A), floR, sul1,*	*AslA, anr, capU, csgA, cvaC, fimH, fyuA, gad, hlyE, hlyF, ireA, iroN, irp2,*	93	O5:H4	IncFIB、IncFIC	Y	N	N

							sul2, tet(A), dfrA12	*iss, iucC, iutA, kpsE, kpsMII_K5, nlpI, ompT, papA, shiA, sitA, terC, tia, traJ, yehA, yehB, yehC, yehD*						
59	23THG141EC(2RESBL)	2023spring	Ningxia	+	CZO-AMP-PIP	2R	*blaCTX-M-1, sitABCD,*	*AslA, chuA, clbB, cnf1, csgA, dhaK, fdeC, fimH, focC, sfaE, fyuA, gad, hha, hlyA, hra, ibeA, ireA, iroN, irp2, iss, kpsE, kpsMII_K1, mchB, mchC, mchF, mcmA, neuC, nlpI, ompT, papA, papC, pic, sfaD, sfaS, shiB, sitA, terC, usp, vat, yehA, yehB, yehC, yehD, yfcV,*	998	O2:H6	ND	Y	Y	N
60	23THY159EC(2RESBL)	2023spring	Ningxia	+	CZO-AMP-PIP	2R	*blaCTX-M-14, sitABCD*	*AslA, chuA, clbB, cnf1, csgA, dhaK, fdeC, fimH, focCsfaE, fyuA, gad, hha, hlyA, hra, ibeA, ireA, iroN, irp2, iss, kpsE, kpsMII_K1, mchB, mchC, mchF, mcmA, neuC, nlpI, ompT, papA_F13, papC, pic, sfaD, sfaS, shiB, sitA, terC, usp, vat, yehA, yehB, yehC, yehD, yfcV*	998	O2:H6	ND	Y	Y	N
61	23THG175EC	2023spring	Ningxia	+	CZO-CTX-FEP-ATM-AMP-PIP	4R	*blaCTX-M-15, qnrS1*	*AslA, air, chuA, csgA, cswA, cswB, cswC, cswE, cswF, cswG, cswR, eilA, eltIAB-18, estap-STa1, fdeC, fimH, gad, hha, hlyE, iss, kpsE, kpsMII, nlpI, ompT, terC, traT, yehA, yehB, yehC, yehD*	38	O7:H18	IncFII	N	N	N
62	23THG240EC	2023spring	Ningxia	-	AMP-SAM-CHL-TET	4R	*aadA1, blaOXA-10, blaTEM-1, sitABCD, cmlA1, floR, qnrS1, ARR-2, tet(A), dfrA14*	*AslA, afaD, anr, chuA, csgA, dhaK, fdeC, fimH, fyuA, hha, ibeA, irp2, kpsE, kpsMII, nlpI, ompT, papC, pic, sitA, terC, traJ, traT, usp, vat, yehA, yehB, yehC, yehD, yfcV*	5261	O18:H7	IncFIB、IncFII	Y	N	N
63	23THY257EC	2023spring	Ningxia	-	GEN-AMP-PIP-SXT-CHL-CIP-LVX-MXF-TET	6R	*aph(6)-Id, aac(3)-IId, aadA2, aph(3")-Ib, blaTEM-1, qacE, sitABCD, mph(A), floR, sul1, sul2, tet(A), dfrA12*	*AslA, anr, capU, csgA, cvaC, fimH, fyuA, gad, hlyE, hlyF, ireA, iroN, irp2, iss, iucC, iutA, kpsE, kpsMII_K5, nlpI, ompT, papA, shiA, sitA, terC, tia, traJ, yehA, yehB, yehC, yehD*	93	O5:H4	IncFIB、IncFIC	Y	N	N
64	23THY275EC	2023spring	Ningxia	+	CZO-CTX-FEP-AMP-PIP	3R	*blaCTX-M-15, qnrS1*	*aalA, aalB, aalF, aalH, aalR, cia, csgA, faeF, faeI, fdeC, fimH, gad, hlyE, lpfA, nlpI, terC, yehA, yehB, yehC, yehD,*	13214	O93:H28	IncFIB、IncI1-I	N	N	Y
65	23THG276EC	2023spring	Ningxia	-	AMP-PIP-SXT-CHL-TET	4R	*aadA1, aadA2, blaTEM-1, qacL, sitABCD, cmlA, qnrB7, sul3, tet(A), dfrA12*	*anr, cma, csgA, cvaC, faeC, fdeC, fimH, gad, hha, hlyE, iroN, iss, lpfA, nlpI, sitA, terC, traJ, traT, yehA, yehB, yehC, yehD*	196	O8:H7	IncFIB、IncFII、IncFII	N	N	N

66	23THG318EC	2023spring	Ningxia	+	GEN-CZO-CTX-FEP-ATM-AMP-PIP-SXT-CHL-TET	8R	*aph(6)-Id, aph(3')-Ia, aac(3)-IId, aadA22, aph(3")-Ib, blaTEM-1, blaCTX-M-55, blaLAP-2, lnu(F), mph(A), cmlA1, floR, qnrS1, sul2, sul3, tet(A), dfrA14*	*AslA, astA, csgA, etsC, fdeC, fimH, fyuA, gad, hha, hlyE, irp2, nlpI, papA, shiA, terC, tia, yehA, yehB, yehC, yehD*	43	O6:H10	IncFIB、IncHI2、IncHI2A	N	N	N
67	NM15	2018spring	Inner Mongolia	-	AMP-PIP-SXT-TET	3R	*aph(6)-Id, aph(3")-Ib, blaTEM-1, sitABCD, mdf(A), sul2, tet(A), dfrA14*	*air, chuA, cvaC, eilA, etsC, gad, hlyF, hra, iroN, iss, iucC, iutA, kpsE, lpfA, mchF, ompT, sitA, terC, traT, tsh, yfcV*	1485	O83:H42	IncFIA、IncFIB、IncFIC、	N	N	N
68	NMB6	2018spring	Inner Mongolia	-	AMP-PIP-SAM-SXT-CHL-TET	5R	*aph(6)-Id, aadA1, aph(3")-Ib, blaOXA-10, blaTEM-1, mdf(A), cmlA1, floR, qnrS1, ARR-2, sul2, tet(A), dfrA14*	*lpfA, terC*	6419	ND	IncY	N	N	N
69	NMJ1	2018spring	Inner Mongolia	-	AMP-AMC-CHL-TET	4R	*aadA1, blaOXA-10, sitABCD, mdf(A), cmlA1, floR, qnrS1, ARR-2, tet(A), tet(A), dfrA14*	*iss, lpfA, ompT, papC, sitA, terC*	783	O83:H9	IncFIB	N	N	N
70	NMJ6	2018spring	Inner Mongolia	-	AMP-PIP-SXT-TET	3R	*aph(6)-Id, aph(3")-Ib, blaTEM-1, mdf(A), qnrS1, sul2, tet(A), dfrA14*	*gad, terC*	48	O5:H11	p0111、IncFIB、	N	N	N
71	NMJ7	2018spring	Inner Mongolia	-	AMP-PIP-SXT-TET	3R	*aph(6)-Id, aph(3")-Ib, blaTEM-1, blaLAP-2, mdf(A), qnrS1, sul2, tet(A), dfrA14*	*gad, terC*	48	O178:H11	p0111	N	N	N
72	YO-3	2019spring	Inner Mongolia	+	CZO-CTX-FEP-AMP-PIP	3R	*blaCTX-M-1, mdf(A)*	*astA, celb, chuA, kpsMII_K5, ompT, terC*	1125	O139:H19	Col156、IncFII、IncI1-I、IncX4	N	N	N
73	YO-5	2019spring	Inner Mongolia		AMP-PIP-SXT-CIP-LVX-MXF-TET	4R	*aph(6)-Id, aph(3")-Ib, blaTEM-1, mdf(A), qnrS1, sul2, tet(A), dfrA14*	*lpfA, terC, traT*	2448	O103:H7	IncFIB、IncFIC	N	N	N
74	YO-7	2019spring	Inner Mongolia		AMP-PIP-SXT-CIP-LVX-MXF-TET	4R	*aph(6)-Id, aph(3")-Ib, blaTEM-1, mdf(A), qnrS1, sul2, tet(A), dfrA14*	*lpfA, terC, traT*	2448	O103:H7	IncFIB、IncFIC、	N	N	N
75	YO-9	2019spring	Inner Mongolia		AMP-PIP-SXT-CIP-LVX-MXF-TET	4R	*aph(6)-Id, aph(3")-Ib, blaTEM-1, mdf(A), qnrS1, sul2, tet(A), dfrA14*	*lpfA, terC, traT*	2448	O103:H7	IncFIB、IncFIC	N	N	N
76	YO-11	2019spring	Inner Mongolia		AMP-PIP-SXT-CIP-LVX-MXF-TET	4R	*aph(6)-Id, aph(3")-Ib, blaTEM-1, mdf(A), qnrS1, sul2, tet(A), dfrA14*	*lpfA, terC, traT*	2448	O103:H7	IncFIB、IncFIC	N	N	N
77	YO-55	2019spring	Inner Mongolia		AMP-PIP-SXT-CIP-LVX-MXF-TET	4R	*aph(6)-Id, aph(3")-Ib, blaTEM-1, mdf(A), qnrS1, sul2, tet(A), dfrA14,*	*lpfA, terC, traT*	2448	O103:H7	IncFIB、IncFIC、	N	N	N
78	NS-E186	2021spring	Inner Mongolia	-	SXT-CHL-TET	3R	*aadA2, qacE, catA2, sul1, sul2, tet(A), dfrA12*	*csgA, fimH, gad, nlpI, terC, yehB, yehC, yehD*	522	O148:H30	IncY	N	N	N
79	NS-E189	2021spring	Inner Mongolia	+	GEN-CZO-CTX-FEP-AMP-PIP-SAM-STX	6R	*aac(3)-IId, aadA5, blaCTX-M-14, blaTEM-1, qacE, sitABCD, mph(A), sul1, dfrA17*	*AslA, aamR, afaD, air, chuA, csgA, eilA, espY2, fdeC, fimH, fyuA, gad, hlyE, hra, irp2, iss, kpsE, kpsMII_K5, nlpI, sitA, terC, traT, yehB, yehC, yehD*	13292	O102:H6	IncFIB	Y	N	N
80	NS-359dc	2021autumn	Inner Mongolia	-	AMP-PIP-SXT-CIP-LVX-MXF-TET	4R	*blaTEM-1, tet(A), sul1, aadA1, qacE*	*AslA, csgA, fimH, gad, hlyE, nlpI, terC, yehA, yehB, yehC, yehD*	4542	ND	IncY	N	N	N
81	NH-190dc	2022spring	Inner Mongolia	-	GEM-AMP-PIP-SXT-TET	4R	*blaTEM-1, tet(A), sul2, aac(3)-IId, aph(6)-Id, aph(3")-Ib, sitABCD, dfrA17*	*AslA, anr, chuA, csgA, eilA, fdeC, fimH, gad, hlyE, hlyF, iroN, iss, iucC, iutA, katP, kpsE, kpsMII, lpfA, nlpI, ompT, sitA, terC, traJ, traT, yehA, yehB, yehC, yehD*	69	ND	IncFIB	Y	N	N

82	NH-195dc	2022spring	Inner Mongolia	-	GEM-AMP-PIP-SXT-TET	4R	*blaTEM-1, tet(A), sul2, aac(3)-IId, aph(6)-Id, aph(3'')-Ib, sitABCD, dfrA17*	*AslA, anr, chuA, csgA, eilA, fdeC, fimH, gad, hlyE, hlyF, iroN, iss, iucC, iutA, katP, kpsE, kpsMII, lpfA, nlpI, ompT, sitA, terC, traJ, traT, yehA, yehB, yehC, yehD*	69	ND	IncFIB	Y	N	N
83	NH-223dc	2022spring	Inner Mongolia	-	GEM-AMP-PIP-SXT-TET	4R	*blaTEM-1, tet(A), sul2, aac(3)-IId, aph(6)-Id, aph(3'')-Ib, sitABCD, dfrA17,*	*AslA, anr, chuA, csgA, eilA, fdeC, fimH, gad, hlyE, hlyF, iroN, iss, iucC, iutA, katP, kpsE, kpsMII, lpfA, nlpI, ompT, sitA, terC, traJ, traT, yehA, yehB, yehC, yehD*	69	ND	IncFIB	Y	N	N
84	22YO-E14	2022autumn	Inner Mongolia	-	ATM-AMC-TET	3R	*tet(A)*	*csgA, fdeC, fimH, gad, hlyE, nlpI, terC, yehA, yehB, yehC, yehD*	609	O9:H4	ND	N	N	N
85	22NH-E50	2022autumn	Inner Mongolia	+	CZO-CTX-FEP-AMP-PIP	3R	*blaCTX-M-1*	*AslA, aalF, aalF, astA, chuA, colE2-like, faeC, faeD, faeE, faeF, faeI, faeJ, fimH, gad, kpsMII_K5, nlpI, ompT, papC, terC, yehB, yehD*	7350	O139:H56	Col156、IncFII、IncI1-I、IncX4	Y	N	N
86	23NH-10EC	2023spring	Inner Mongolia	+	CZO-CTX-FEP-AMP-PIP-AMC-SAM-SXT-CHL-TET	7R	*aph(6)-Id_1_M28829, aph(3'')-Ib, blaCTX-M-14, blaTEM-1A, sitABCD, floR, sul2, tet(A), dfrA14*	*AslA, air, anr, chuA, csgA, cvaC, eilA, espY2, etsC, fdeC, fimH, gad, hlyE, hlyF, hra, iroN, iss, iucC, iutA, kpsE, kpsMII_K5, lpfA, mchF, mchF, mcmA, nlpI, ompT, sitA, terC, traT, tsh, yehB, yehC, yehD, yfcV*	1485	O83:H42	IncFIA、IncFIB、IncFIC、IncI1-I、IncX1	Y	N	Y
87	23NH-39EC	2023spring	Inner Mongolia	-	PIP-AMC-TET	3R	*blaTEM-1, sitABCD, tet(A)*	*AslA, anr, astA, cdt-IVB, chuA, cia, csgA, cvaC, dhaK, etsC, fdeC, fimH, fyuA, gad, hlyF, ibeA, iroN, irp2, iss, iucC, iutA, kpsE, kpsMII_K5, mchF, nlpI, ompT, papC, sitA, terC, tia, traJ, traT, usp, yehA, yehB, yehC, yehD, yfcV*	131	O25:H4	IncFIB、IncFII、IncX1	N	Y	N
88	23NH-53EC	2023spring	Inner Mongolia	-	AMP-PIP-SXT-CIP-LVX-MXF-TET	4R	*aph(6)-Id, aph(3'')-Ib, blaTEM-1, sitABCD, fosA7, sul2, tet(A), dfrA14*	*anr, csgA, cvaC, etsC, fdeC, fimH, gad, hlyE, hlyF, iroN, iss, iucC, iutA, lpfA, mchF, nlpI, ompT, shiB, sitA, terC, traJ, traT, yehA, yehB, yehC, yehD*	602	ND	IncFIB、IncFIC	N	N	N
89	23YO-34EC	2023spring	Inner Mongolia	-	AMP-PIP-SXT-CIP-LVX-MXF-TET	4R	*aph(6)-Id, aph(3'')-Ib, blaTEM-1, sitABCD, fosA7, sul2, tet(A), dfrA14*	*anr, csgA, cvaC, etsC, fdeC, fimH, gad, hlyE, hlyF, iroN, iss, iucC, iutA, lpfA, mchF, nlpI, ompT, shiB, sitA, terC, traJ, traT, yehA, yehB, yehC, yehD*	ND	ND	ND	N	N	N
90	23YO-120EC	2023spring	Inner Mongolia		AMP-PIP-SXT-CHL-TET	4R	*aph(3')-Ia, blaTEM-176, floR, qnrS1, tet(A), dfrA14*	*csgA, cvaC, ehxA, fdeC, fimH, gad, hha, hlyE, iss, lpfA, nlpI, ompT, terC, tia, traT, yehA, yehB, yehC, yehD*、	295	ND	IncFIB、IncFII、IncX1、	N	N	N

91	23YO-162EC	2023spring	Inner Mongolia	GEN-CZO-CTX-FEP-AMP-PIP-SXT-CHL-TET	7R	*aadA5, aph(4)-Ia, aac(3)-IV, blaCTX-M-14, blaTEM-1, fosA3, mph(A), floR, qnrS1, sul2, tet(A), dfrA17*	*csgA, fdeC, fimH, gad, hha, hlyE, iss, lpfA, nlpI, papC, terC, yehA, yehB, yehC, yehD,*	5552	O54:H28	IncFIB、IncHI2、IncHI2A	N	N	Y

ND: Not determined; Abbreviations: AMC, Amoxicillin-Clavulanate; AMP, Ampicillin; ATM, Aztreonam; CAZ, Ceftazidime; CHL, Chloramphenicol; CIP, Ciprofloxacin; CTX, Cefotaxime; CZO, Cefazolin; FEP, Cefepime; GEN, Gentamicin; LVX, Levofloxacin; MXF, Moxifloxacin; PIP, Piperacillin; SXT, Trimethoprim-Sulfamethoxazole; TET, Tetracycline.
